# Shigella IpaD has a dual role: signal transduction from the type III secretion system needle tip and intracellular secretion regulation

**DOI:** 10.1111/mmi.12124

**Published:** 2013-01-11

**Authors:** A Dorothea Roehrich, Enora Guillossou, Ariel J Blocker, Isabel Martinez-Argudo

**Affiliations:** 1 School of Cellular & Molecular Medicine, University of BristolUniversity Walk, Bristol, BS8 1TD, UK; 2 School of Biochemistry, University of BristolUniversity Walk, Bristol, BS8 1TD, UK

## Abstract

Type III secretion systems (T3SSs) are protein injection devices essential for the interaction of many Gram-negative bacteria with eukaryotic cells. While *Shigella* assembles its T3SS when the environmental conditions are appropriate for invasion, secretion is only activated after physical contact with a host cell. First, the translocators are secreted to form a pore in the host cell membrane, followed by effectors which manipulate the host cell. Secretion activation is tightly controlled by conserved T3SS components: the needle tip proteins IpaD and IpaB, the needle itself and the intracellular gatekeeper protein MxiC. To further characterize the role of IpaD during activation, we combined random mutagenesis with a genetic screen to identify ipaD mutant strains unable to respond to host cell contact. Class II mutants have an overall defect in secretion induction. They map to IpaD's C-terminal helix and likely affect activation signal generation or transmission. The Class I mutant secretes translocators prematurely and is specifically defective in IpaD secretion upon activation. A phenotypically equivalent mutant was found in mxiC. We show that IpaD and MxiC act in the same intracellular pathway. In summary, we demonstrate that IpaD has a dual role and acts at two distinct locations during secretion activation.

## Introduction

Type III secretion systems (T3SSs) are common virulence factors among Gram-negative bacteria. They are protein transport devices used for injecting effector proteins into host cells to modulate their responses in favour of the bacterium (Cornelis, 2006). To study conserved aspects of type III secretion, we use *Shigella flexneri* as a model system. It is the causative agent of human bacillary dysentery, a form of inflammatory diarrhoea, and uses the T3SS to promote its invasion into gut epithelial cells. The T3SS consists of a basal body spanning both bacterial membranes and a hollow extracellular needle serving as secretion channel. The distal end of the needle is topped by the tip complex, which is required for pore formation and protein translocation into host cells. In *Shigella*, the apparatus is assembled when the environmental conditions are appropriate for invasion, but secretion is blocked until physical contact with a host cell generates an activation signal (reviewed in Schroeder and Hilbi, 2008).

Protein translocation into host cells requires three proteins known as translocators (IpaB, IpaC and IpaD in *Shigella*). Of these, IpaD is hydrophilic and constitutively present atop mature needles (Espina *et al*., 2006; Sani *et al*., 2007; Veenendaal *et al*., 2007). It probably serves as a scaffold for the mostly hydrophobic proteins IpaB and IpaC, which later form the pore in the host cell membrane. IpaB is also constitutively present at needle tips, while the other hydrophobic translocator, IpaC, is only recruited to the needle tip upon activation (Veenendaal *et al*., 2007; Roehrich *et al*., 2010; Shen *et al*., 2010). In *Yersinia*, the needle tip is formed by the hydrophilic translocator LcrV: in this species no hydrophobic translocator has been found constitutively present atop needles (Mueller *et al*., 2005; 2008; Blocker *et al*., 2008).

Type III secretion is a tightly regulated process initiated only after host cell contact. As type III proteins are expressed when the environmental conditions are appropriate for invasion, secretion has to be prevented prior to cell contact. Recently, we proposed a model featuring gating mechanisms located in the cytoplasm that prevent secretion in the absence of activation signals (Martinez-Argudo and Blocker, 2010). We hypothesized that under non-activated conditions, a mechanism that represses translocator secretion, directly or indirectly involving IpaD and/or IpaB, is put in place. In addition, the gatekeeper protein MxiC prevents premature effector secretion (Botteaux *et al*., 2009; Martinez-Argudo and Blocker, 2010) and it requires a secretion signal to do so (Botteaux *et al*., 2009). After reception of the activation signal, such repression mechanisms would be counteracted and secretion happens in an ordered way: translocator proteins are secreted first, then MxiC (Kenjale *et al*., 2005; Martinez-Argudo and Blocker, 2010) followed by early and late effectors (Mavris *et al*., 2002; Parsot, 2009). As a Δ*mxiC* mutant shows weak and delayed secretion of translocators in response to activation when compared with the wild-type strain, we suggested that, in *Shigella*, MxiC also plays a role in activating translocator secretion (Martinez-Argudo and Blocker, 2010).

MxiC belongs to a family of T3SS proteins that includes *Yersinia* YopN/TyeA, enteropathogenic *Escherichia coli* SepL and *Salmonella* InvE and SsaL (Pallen *et al*., 2005). Besides similar sequences, the proteins have conserved domain topologies and structures (Schubot *et al*., 2005; Deane *et al*., 2008; 2010) and all block effector secretion in the absence of an activation signal. However, mutants do not show the same phenotype with respect to the control of translocator secretion. In Δ*invE*, Δ*ssaL*, Δ*sepL* and Δ*mxiC* mutants, secretion of translocators is impaired (Kubori and Galan, 2002; Coombes *et al*., 2004; O'Connell *et al*., 2004; Deng *et al*., 2005), whereas it is constitutive in Δ*yopN* or Δ*tyeA* mutants (Iriarte *et al*., 1998; Ferracci *et al*., 2005). How the gatekeepers perform their tasks is unclear, but *Salmonella* InvE interacts with chaperone–translocator complexes (Kubori and Galan, 2002) and *Shigella* MxiC interacts with the ATPase *in vitro* (Botteaux *et al*., 2009).

Despite intense research we still do not understand how the activation signal generated after contact with the host cell is transduced to the cytoplasmic side of the T3SS to initiate secretion. The first part of the T3SS that establishes contact with the host cell is probably the needle tip. Therefore, it is likely that the tip complex senses the host cell. Experimental data from the *Shigella* and *Yersinia* systems suggest that the needle is directly involved in transmitting the activation signal. Particular single amino acid mutations in the needle protein lead to altered secretion phenotypes and tip complex composition (Kenjale *et al*., 2005; Torruellas *et al*., 2005; Veenendaal *et al*., 2007).

The *Shigella* needle protein MxiH is essentially a coiled coil hairpin that polymerizes into the helical needle using both of its termini (Cordes *et al*., 2003; Kenjale *et al*., 2005; Deane *et al*., 2006; Fujii *et al*., 2012; Loquet *et al*., 2012). Similar to the needle protein MxiH, IpaD contains a longer central coiled coil and requires its extreme C-terminus for binding to the needle. In addition, it has two globular domains, one at the N-terminus and one linking the two helices of the coiled coil (Johnson *et al*., 2007; Veenendaal *et al*., 2007). The C-terminal globular domain is a site of interaction with IpaB, while the N-terminal globular domain of IpaD has been proposed to act as a self-chaperone (Johnson *et al*., 2007). In the LcrV class of hydrophilic tip proteins, the chaperone is formed by a separate polypeptide called LcrG (Lawton *et al*., 2002).

The needle tip was first visualized in the *Yersinia* T3SS (Mueller *et al*., 2005). We proposed that the tip contains four molecules of IpaD and one molecule of IpaB (Johnson *et al*., 2007; Veenendaal *et al*., 2007; Blocker *et al*., 2008). The crystal structure of IpaD allowed construction of a pentamer based on dimer contacts in one of the crystal forms with a helical rise similar to that of the MxiH needle (Cordes *et al*., 2003; Johnson *et al*., 2007). In the proposed IpaD pentamer, the position between the fourth and first IpaD molecules is structurally different from all other positions. This final position might thus be filled by IpaB (Johnson *et al*., 2007; Veenendaal *et al*., 2007; Blocker *et al*., 2008). The model puts the proposed central coiled coil and the C-terminal globular domain of IpaB in an equivalent conformation to that of IpaD, positioning IpaB's domain homologous to pore-forming colicins and its putative transmembrane helices in the ideal place to interact with the host cell membrane (Johnson *et al*., 2007; Barta *et al*., 2012a). Together with mutagenesis data this suggests that IpaB could be the host cell sensor and that a conformational change in the tip complex might be signalling event triggering T3SS activation (Veenendaal *et al*., 2007; Roehrich *et al*., 2010; Shen *et al*., 2010).

Congo red (CR) is a small amphipathic dye molecule that was originally used to assess infectivity of *Shigella* strains: only colonies binding CR on agar plates were infective, while ‘white’ colonies were not (Payne and Finkelstein, 1977). It was later found that CR specifically activates type III secretion (Parsot *et al*., 1995) and that its target is likely in the tip complex (Veenendaal *et al*., 2007). All T3SS component-knockout (KO) strains that are not inducible by CR are also non-invasive, suggesting that CR and host cell contact induce T3SS activation using a related mechanism (Menard *et al*., 1993; 1994; Blocker *et al*., 2001).

To test our hypothesis that, as part of the tip complex, IpaD is involved in conformational changes crucial for signal transduction, we sought to identify *ipaD* point mutants unable to respond to host cell contact. For ease of analysis, we chose to set up a genetic screen for mutants insensitive to induction by CR. All mutants isolated showed a wild-type-like tip complex composition. Yet, six out of seven mutants were impaired in host cell sensing and induction of type III secretion. The position of these mutations highlights the importance of the C-terminal helix of IpaD in signal transduction. The remaining *ipaD* mutant was unable to prevent secretion under non-inducing conditions. Parallel analysis of a *mxiC* mutant with a similar phenotype further supports the regulatory roles of both IpaD and MxiC in translocator secretion. Taken together, these data demonstrate that IpaD has a dual role during secretion activation.

## Results

For the *Shigella* T3SS, ‘induction’ describes the burst of Ipa protein secretion upon host cell sensing (Menard *et al*., 1994) or addition of CR, a small amphipathic dye molecule which acts as an artificial inducer of its T3SS (Bahrani *et al*., 1997). Induction is a very fast event as around 50% of the Ipa proteins are secreted at 37°C in the 15 min following CR addition (Bahrani *et al*., 1997; Magdalena *et al*., 2002; Parsot *et al*., 2005). Prior to induction there is a slow, low level of Ipa protein secretion known as ‘leakage’, where around 5% of Ipa proteins are secreted (Magdalena *et al*., 2002; Parsot *et al*., 2005). ‘Constitutive secretion’ of Ipa proteins represents an unregulated and higher-level secretion than leakage and involves not only the Ipa proteins but also the ‘late effectors’, which are involved in later stages of infection. In the Δ*ipaB* and Δ*ipaD* mutant strains, constitutive secretion is very fast and detectable in minutes. It is thus named ‘fast constitutive secretion’ (Veenendaal *et al*., 2007). We recently showed that constitutive secretion has a different cause in a Δ*ipaB* versus a *ipaB*Δ*3* mutant (Martinez-Argudo and Blocker, 2010). As the latter mutant is in a constitutively activated state, we now call its phenotype ‘premature secretion’.

### Isolation of ipaD mutants affected in secretion

To test our hypothesis that IpaD is involved in the reception or transduction of the activation signal, we sought to identify *ipaD* mutant strains that are unable to secrete after induction by CR. For this, we used a screening method based on the colour of *Shigella* colonies in plates containing CR. Wild-type *Shigella* colonies stain light red on CR-containing plates, while mutants unable to secrete effectors are white (Maurelli *et al*., 1984). Constitutive secretor mutant colonies, such as those containing bacteria that have lost their needle tip, are bright red on CR plates, probably because of the secretion of late effectors (Parsot *et al*., 1995). Upon random PCR mutagenesis of the *ipaD* gene, the mutant DNA libraries were cloned and amplified as described in *Experimental procedures*. After transformation of the mutant libraries into the *Shigella* Δ*ipaD* mutant, we screened for white colonies in plates containing CR (Fig. S1). Around 500 000 transformants were screened and as expected a substantial proportion of them were bright red (10–20%, indicating expression of non-functional *ipaD* alleles). One hundred and ten ‘white’ colonies were selected and their plasmids isolated and retransformed into the Δ*ipaD* mutant to ensure the white colour was not due to loss-of-function mutations elsewhere within the T3SS-encoding operons.

Twenty-two plasmids were confirmed as giving rise to white colonies and their *ipaD* gene was sequenced (Table 1). Mutant *ipaD(L99P, N255D)* was not white but slightly more red than wild-type when retransformed into the Δ*ipaD* mutant; however, it showed an interesting phenotype (see below) and was further investigated. In cases where more than one mutation was found in the *ipaD* gene, individual mutations were cloned independently in order to identify which was responsible for the mutant phenotype and only the mutation generating the mutant phenotype was further investigated.

**Table 1:** *ipaD* mutants isolated in this study and their phenotypes

Class	*ipaD* mutation	Times the mutant was found	Phenotypic properties				
			Leakage	CR induction	IpaD expression	Needle tip composition	Invasion
–	Wild-type	–	WT	WT	WT	WT	WT
–	Knockout	–	++	(–)^a^	NA	– –	– –
I	***L99P***, *N255D*	1	+	(WT)^b^	–	WT	WT
IIa	***N186Y***	1	WT	– –	WT	WT	–
IIa	***N273I***, ***Q277L***	1	WT	– –	WT	WT	–
IIa	***K291I*** *(or T or N)*	7^c^	WT	– –	WT	WT	–
IIb	***N292Y***	1	WT	–	WT	WT	WT
IIb	***T296I***	11^c^	WT	–	WT	WT	–
IIb	*Y104L*, ***Q299L***	1	WT	–	WT	WT	–

The mutation responsible for the observed phenotype is indicated in bold letters. ‘WT’ indicates wild-type-like properties, + or ++ an increase and – or – – a decrease. ‘NA’ means not applicable.

a Δ*ipaD* is not inducible by CR, but nevertheless proteins are detectable as it is a fast constitutive secreter.

b Mutant L99P only has a defect in IpaD secretion after CR induction.

c Mutants K291I/T/N and T296I were found independently in two and three separate libraries respectively.

### *ipaD* mutant strains have differentially altered secretion profiles

**Figure 1:**
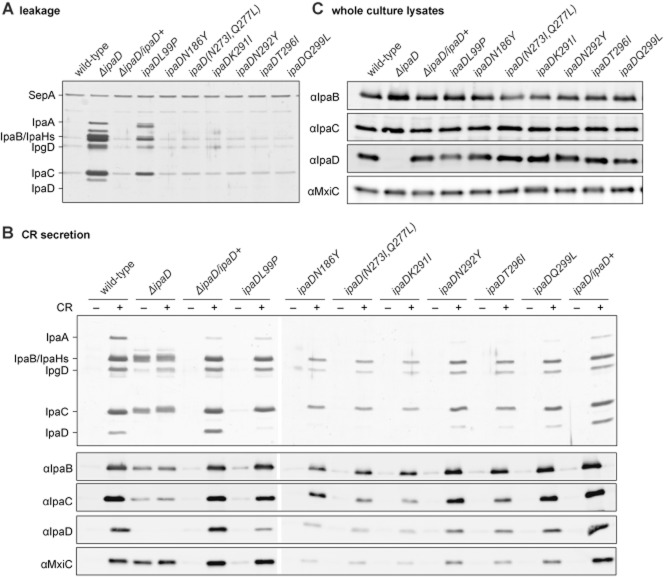
All *ipaD* mutants isolated cause partial defects in secretion. A. Exponential leakage of *Shigella* wild-type, Δ*ipaD* mutant, complemented strain (Δ*ipaD/ipaD*^+^) and *ipaD* mutants (in the Δ*ipaD* background) isolated from a mutant library. Samples were collected as described in *Experimental procedures* and Silver-stained. B. Protein secretion in response to artificial inducer Congo red (CR) induction. Samples were collected as described in *Experimental procedures* and Silver-stained (top panel) and Western-blotted with antibodies against the translocator proteins IpaB, IpaC, IpaD and the regulator MxiC (bottom panels). C. Total protein expression levels. Samples were collected as described in *Experimental procedures* and Western-blotted with the indicated antibodies. Results shown are representative of at least two independent experiments.

To characterize the *ipaD* mutants, we analysed their secretion profile during exponential growth in non-inducing conditions (Fig 1A) and following CR induction (Fig 1B). We found two novel types of secretion profile. First, mutant *ipaDL99P* secreted a much higher level of T3S proteins in non-inducing conditions when compared to the Δ*ipaD/ipaD*^+^ complemented strain, but still less than the Δ*ipaD* mutant (Fig 1A, *c.* 4 h incubation). Additionally, mutant *ipaDL99P* showed a defect in IpaD secretion (approximately 20% of the complemented strain) following CR induction, while secretion of IpaB, IpaC, MxiC and effectors was still induced to levels comparable with the complemented strain (Fig 1B). In this 15 min assay, increased secretion under non-inducing conditions is only visible if it is ‘fast’ as for example in the Δ*ipaD* mutant. In a second group of mutants (*ipaDN186Y*, *ipaDK291* and *ipaD(N273I, Q277L)*), induction of secretion was impaired: in comparison to the Δ*ipaD/ipaD*^+^ complemented strain they secreted only approximately 40%, 20%, 10% and 10% of IpaB, IpaC, IpaD and MxiC respectively. Mutants *ipaDN292Y*, *ipaDT296I* and *ipaDQ299L* showed a similar secretion pattern, although the defects in secretion were less pronounced (approximately 80%, 60%, 40% and 20% of IpaB, IpaC, IpaD and MxiC respectively; Fig 1B). We named these Classes I, IIa and IIb respectively, starting the numbering with the most N-terminal mutation. When we analysed the expression of the IpaD mutant proteins, the level of the mutant proteins was similar to the complemented strain with the exception of the IpaDL99P, which was present at around 60% of the wild-type allele (Fig 1C). As deletion of *ipaD* leads to constitutive secretion, we were concerned that the increased secretion in *ipaDL99P* under non-inducing conditions might be due to a decrease in IpaD expression. However, the *ipaDL99P* mutant secreted more IpaB and IpaC than a strain expressing much lower levels of the wild-type IpaD protein, indicating that constitutive secretion has a different cause in *ipaDL99P* (Fig. S2). Therefore, all IpaD mutants isolated are partially defective in secretion regulation.

### All IpaD mutant proteins are secreted in a constitutive secretor background

As all the newly isolated *ipaD* mutants showed a defect in IpaD secretion, we wondered if the mutations made them ‘unfit’ for secretion, for example by affecting their conformation in the cytoplasm. To test this possibility we checked if the IpaD mutants were secreted in a constitutive secretor background. We and others have previously shown that the translocator proteins IpaC and IpaD are secreted constitutively in a Δ*ipaB* background (Menard *et al*., 1994; Martinez-Argudo and Blocker, 2010). We generated a Δ*ipaB* Δ*ipaD* double KO strain and after transforming all the *ipaD* mutant plasmids individually into this strain we compared their secretion phenotype to that of the KO strain transformed with a wild-type copy of the *ipaD* gene. Analysis of the secretion profile by Western blot established that the IpaD mutant proteins were constitutively secreted by the Δ*ipaB* Δ*ipaD/ipaD* mutant strains, as efficiently as wild-type IpaD was secreted by the control strain Δ*ipaB* Δ*ipaD/ipaD*^+^ (Fig. S3). This indicates that our mutations are not impairing IpaD's ability to be secreted by the T3SS.

### Class II *ipaD* mutants show defects in invasion of cultured cells

**Figure 2:**
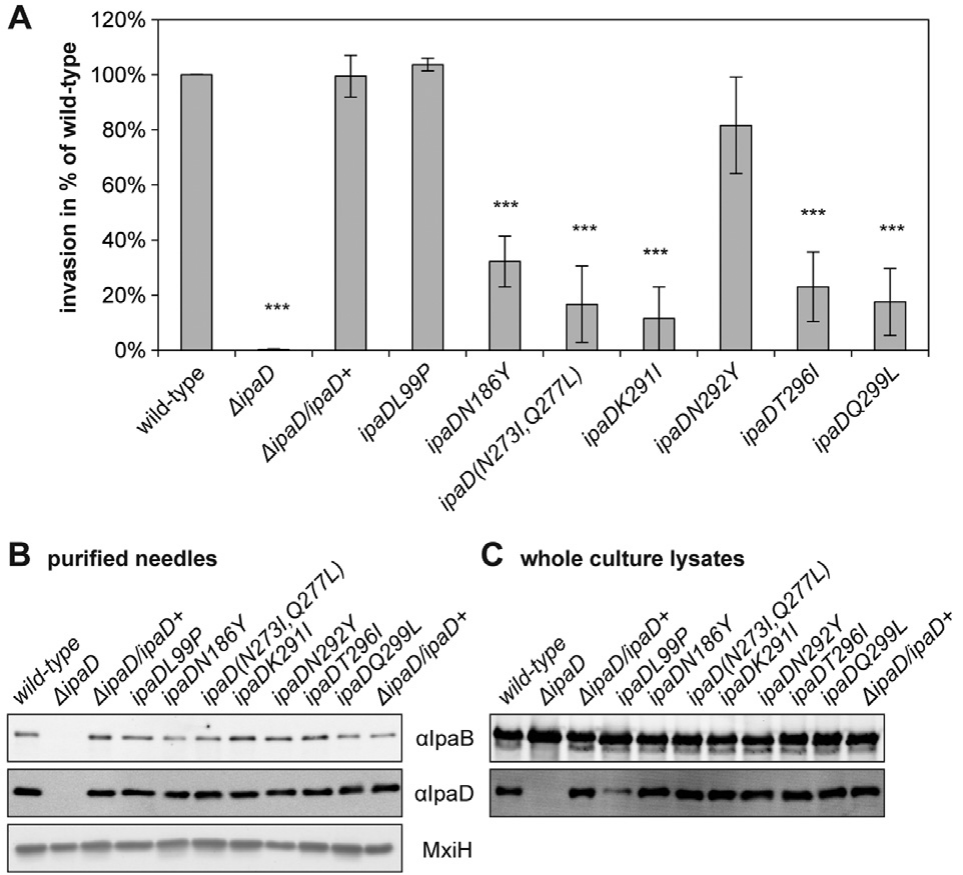
The *ipaD* mutants show defects in HeLa invasion but have normal needle tip compositions. A. Invasion of HeLa cells was measured with a gentamicin protection assay as described in *Experimental procedures*. Every experiment was normalized to wild-type and the data are averages of at least three independent experiments performed at least in duplicate. Error bars indicate the standard deviation. Where the invasion is significantly different from the complemented strain Δ*ipaD/ipaD*^+^ (*P* < 0.001, ANOVA with Tukey's post hoc test), *ipaD* mutant strains (in the Δ*ipaD* background) are marked with ***. B. Needles from wild-type, Δ*ipaD* mutant, complemented strain (Δ*ipaD/ipaD*^+^) and *ipaD* mutants (in the Δ*ipaD* background) overexpressing the needle protein MxiH were isolated as described in *Experimental procedures* and Western-blotted with antibodies against IpaB and IpaD (samples were normalized for the amount of MxiH present in preparations as detected by Silver stain, bottom panel). Results shown are representative of at least two independent experiments. For the complemented strain (Δ*ipaD/ipaD*^+^) results from two experiments are shown to indicate experimental variability. In comparison to the complemented strain, the wild-type contains *c.* 50 ± 10% IpaD (average ± standard deviation), *ipaDL99P* contains *c.* 70 ± 30% IpaD, Class IIa mutants contain *c.* 90 ± 20% IpaD and Class IIb mutants contain *c.* 90 ± 40% IpaD. For IpaB, these values are 70 ± 40% for wild-type, 100 ± 70% for *ipaDL99P*, 150 ± 100% for Class IIa mutants and 130 ± 90% for Class IIb mutants. There are no significant differences between samples in an ANOVA (*P* > 0.05) for both IpaD and IpaB. C. Total protein expression levels in overnight cultures from strains overexpressing the needle protein as in B. Samples were collected as described in *Experimental procedures* and Western-blotted with antibodies against IpaB and IpaD.

To test whether the defect in protein secretion after addition of CR in the *ipaD* mutants correlates with a defect in signal reception/transduction after host cell contact, we analysed whether the *ipaD* mutants are still able to invade HeLa cells. Gentamicin protection assays were performed as described in *Experimental procedures*. In this assay, the Δ*ipaD* mutant is completely non-invasive (Menard *et al*., 1993). All Class II *ipaD* mutant strains, except for *ipaDN292Y*, showed a severe defect in invasion (*c.* 20% of the complemented strain, Fig 2A). Mutant *ipaDL99P* was as invasive as the complemented strain. These results indicate that most of the mutations identified in *ipaD* that cause a defect in CR-induced secretion lead to a defect in invasion of cultured cells.

### *ipaD* mutant strains have normal needle tip compositions

As all isolated *ipaD* mutants had a partial defect in secretion and as IpaD forms part of the tip complex, one possible explanation is that the mutants have a defect in the needle tip composition. Altered tip compositions are known to alter secretion regulation (Veenendaal *et al*., 2007). Therefore, we examined the tip complex composition of the *ipaD* mutants. Needles were purified from the different *ipaD* mutant strains as described in *Experimental procedures* and analysed by Western blot for the presence of the tip proteins IpaD and IpaB. All mutants had wild-type levels of both IpaD and IpaB within the levels of experimental variability (Fig 2B). This was also the case in the *ipaDL99P* mutant, which was expressed at lower levels (Fig 2C). This indicates that none of the mutants has a defect in needle tip complex composition.

### Class IIa and IIb *ipaD* mutants are affected in signalling

**Figure 3:**
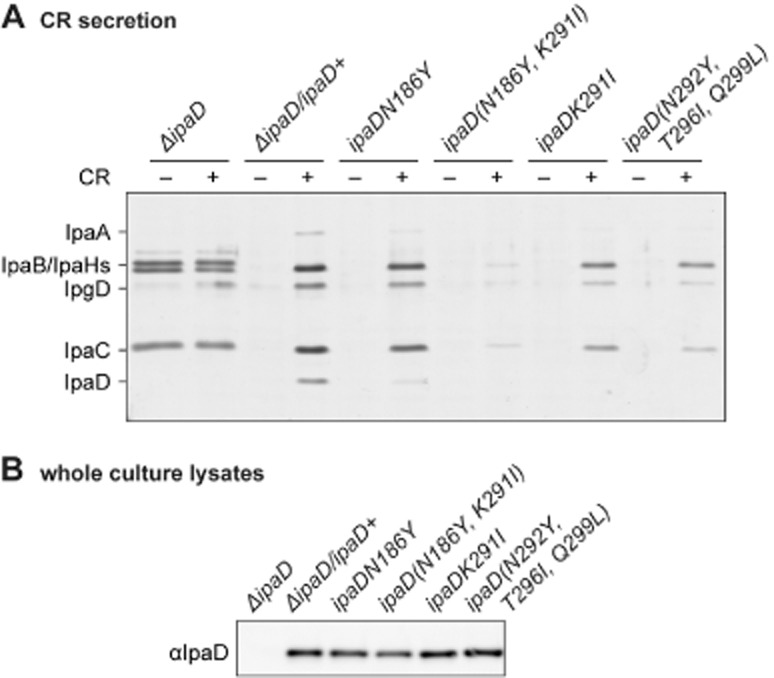
Class II *ipaD* mutants are severely affected in induction of secretion. A. Analysis of proteins secreted by the indicated strains in response to Congo red (CR) induction. Samples from *Shigella* wild-type, Δ*ipaD* mutant, complemented strain (Δ*ipaD/ipaD*^+^) and *ipaD* mutants (in the Δ*ipaD* background) were collected as described in *Experimental procedures* and Silver-stained. B. Total protein expression levels. Samples were collected as described in *Experimental procedures* and Western-blotted with an antibody against IpaD. Results shown are representative of at least two independent experiments.

Induction of secretion was impaired in mutants from Classes IIa and IIb. We have hypothesized that, as part of the tip complex, IpaD is involved in conformational changes essential for the transduction of the activation signal (Veenendaal *et al*., 2007; Martinez-Argudo and Blocker, 2010). Therefore, we should be able to isolate mutants unable to transmit the signal. However, no mutant was completely uninducible. To test whether the combination of mutations would produce a stronger phenotype, we constructed two new mutant *ipaD* plasmids combining Class IIa mutations *N186Y* and *K291I* or Class IIb mutations *N292Y*, *T296I* and *Q299L*. Mutant *ipaD(N186Y, K291I)* considerably reduced the secretion levels following CR induction compared to single N186Y and K291I mutations, while the triple mutant *ipaD(N292Y, T296I, Q299L)* showed a phenotype similar to Class IIa single mutants (Fig 3). Just like the original single mutants, these combinations showed normal leakage (not shown).

Our previous results indicate that the gatekeeper protein MxiC facilitates secretion of the translocators IpaB, IpaC and IpaD, as translocator secretion is only very weakly inducible in a Δ*mxiC* mutant (Martinez-Argudo and Blocker, 2010). We also found that translocator secretion is MxiC-independent in a Δ*ipaB* or Δ*ipaD* constitutive secretor background, but that it is still MxiC-dependent in a constitutively ‘on’ *ipaB*Δ*3* strain, as in the wild-type strain (Martinez-Argudo and Blocker, 2010; Roehrich *et al*., 2010). In the *ipaB*Δ*3* mutant IpaB is expressed lacking its last three C-terminal amino acids. This mutant is locked in a secretion ‘on’ conformation (i.e. similar to the secretion activated wild-type), as it is a constitutive secretor, invasive and recruits some IpaC to the needle tip (Roehrich *et al*., 2010). To rule out the possibility that the decreased secretion of Class II IpaD mutant proteins was due to lack of regulation by MxiC, we investigated if they were secreted in an *ipaB*Δ*3* background. We made a plasmid expressing both *ipaB*Δ*3* and *ipaD* into which all the mutations were subsequently cloned. All plasmids were transformed into the strain Δ*ipaB* Δ*ipaD* and their secretion profile analysed. For mutants belonging to Class II, secretion of IpaD became constitutive in all the mutants (not shown). This was also the case when non-inducible mutant *ipaD(N186Y, K291I)* was expressed in this background. Thus, impaired secretion of these IpaD mutant proteins is not due to a lack of regulation by MxiC. Moreover, the constitutive secretion pattern of all the strains was similar to that of the *ipaB*Δ*3* Δ*ipaD/ipaD*^+^ strain (not shown), indicating that the *ipaB*Δ*3* mutation is epistatic over the *ipaD* Class II mutations.

These results, together with the fact that the composition of the needle tip is wild-type-like for those mutants (Fig 2B), indicate that Class IIa and IIb mutants are affected in signalling from the needle tip and not in a downstream activation step.

### Lack of secretion of the Class I IpaDL99P mutant protein is due to lack of regulation by MxiC

**Figure 4:**
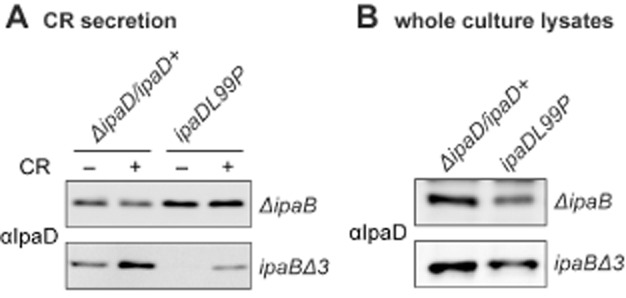
Impaired secretion of IpaDL99P is due to lack of regulation by MxiC. A. Secretion of IpaD after Congo red (CR) induction by a Δ*ipaB* Δ*ipaD* double mutant complemented with either *ipaD* alone or a combined *ipaB*Δ*3 ipaD* plasmid. Samples were collected as described in *Experimental procedures* and Western-blotted with an antibody against IpaD. B. Total protein expression levels. Samples were collected as described in *Experimental procedures* and Western-blotted with an antibody against IpaD. Results shown are representative of at least two independent experiments.

In the *ipaDL99P* mutant strain, only IpaD secretion was impaired after activation (Fig 1B). As mentioned above, secretion of the translocator proteins from the cytoplasm is promoted by MxiC in wild-type and *ipaB*Δ*3* backgrounds (Martinez-Argudo and Blocker, 2010). In order to examine if secretion of the IpaDL99P mutant protein was still dependent on MxiC, we analysed its secretion in the *ipaB*Δ*3* background. When compared with *ipaB*Δ*3* Δ*ipaD/ipaD*^+^, strain *ipaB*Δ*3 ipaDL99P* showed a different pattern of secretion: IpaDL99P secretion was impaired in the *ipaB*Δ*3* background, but constitutive in the Δ*ipaB* background (Fig 4). Therefore, the impairment in IpaDL99P secretion is probably due to a lack of intracytoplasmic regulation by MxiC.

### IpaD has a dual role in T3SS regulation

**Figure 5:**
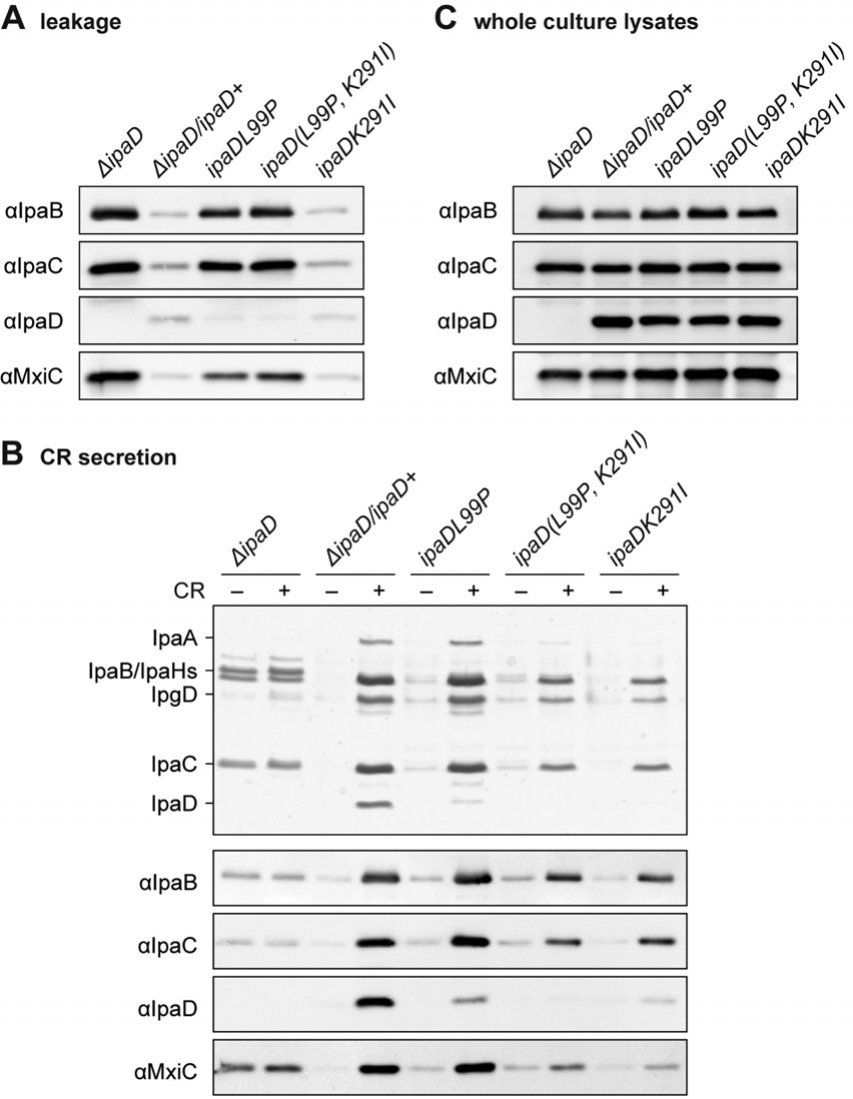
The mutations *L99P* and *K291I* affect different IpaD functions. A. Exponential leakage of the Δ*ipaD* mutant, complemented strain (Δ*ipaD/ipaD*^+^) and *ipaD* mutants (in the Δ*ipaD* background). Samples were collected as described in *Experimental procedures* and Western-blotted with the indicated antibodies. B. Protein secretion in response to Congo red (CR) induction. Samples were collected as described in *Experimental procedures*, Silver-stained (top panel) and Western-blotted with the indicated antibodies (bottom panels). C. Total protein expression levels. Samples were collected as described in *Experimental procedures* and Western-blotted with the indicated antibodies. Results shown are representative of at least two independent experiments.

The *ipaDL99P* strain secretes higher levels of T3S proteins in non-inducing conditions compared to the complemented strain (Fig 1A). To further investigate this, we analysed the supernatant of exponential cultures under non-induced conditions by Western blot and found that the mutant secreted IpaB, IpaC, MxiC and effector proteins (Fig 5A and not shown). This suggested that in the *ipaDL99P* mutant a regulatory function of IpaD is altered and that, as a result, the mechanism that prevents secretion before activation signal reception is impaired. On the other hand, Class II mutants did not show a defect in the prevention of secretion under non-induced conditions (Fig 1A). Therefore, Class I and Class II mutants could affect two different aspects of IpaD function. To test this hypothesis, we made a double mutant combining mutation *ipaDK291I* (Class IIa) and *ipaDL99P* (Class I). If different functions were affected in these classes, one would expect to see both phenotypes in the combined mutant. However, if both classes affected the same function, dominance of one of the phenotypes would be more likely. The mutant *ipaD(L99P, K291I)* showed both phenotypes: it secreted IpaB, IpaC and MxiC under non-inducing conditions and was impaired in CR induction (Fig 5). Together with the fact that secretion in the different mutant classes is differentially affected by an additional *ipaB*Δ*3* mutation, this result suggests that IpaD has a dual role in regulation of T3SS secretion.

### IpaD and MxiC are both involved in the regulation of the mechanism that controls secretion

**Figure 6:**
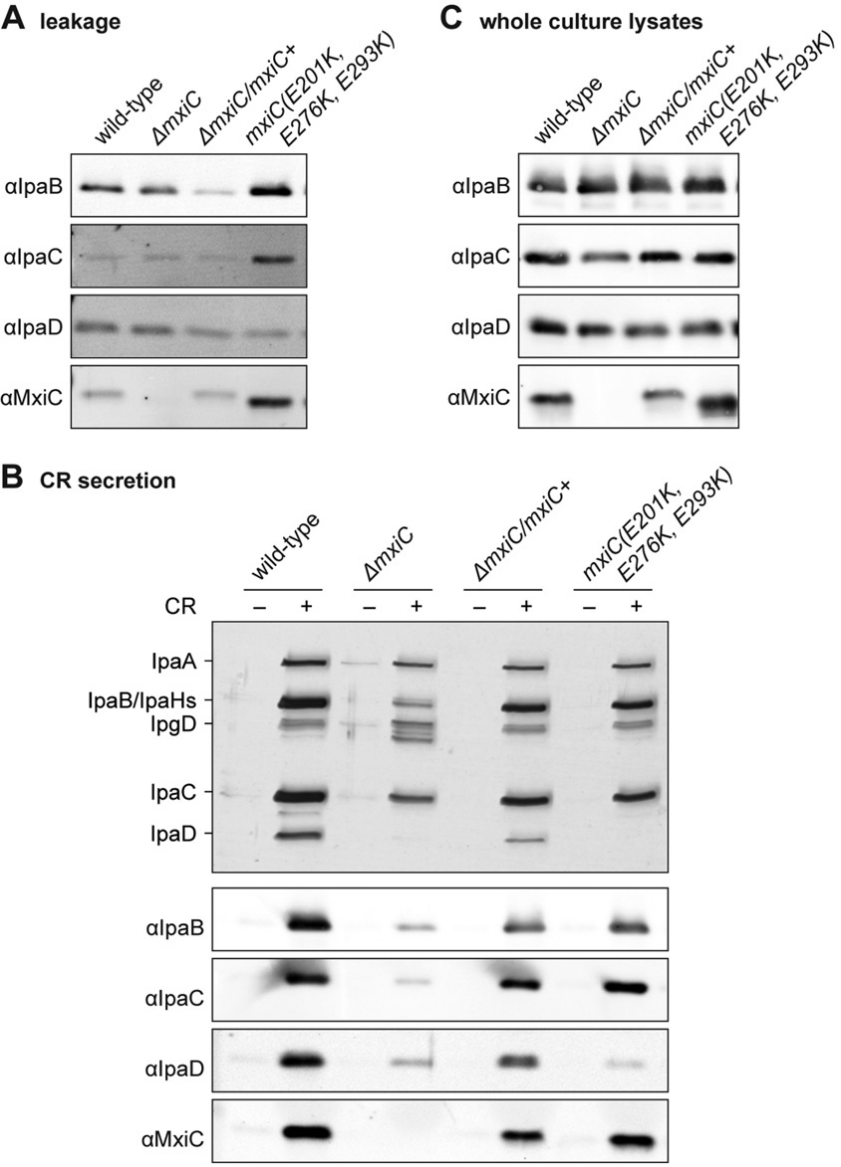
*mxiC(E201K, E276K, E293K)* has a phenotype similar to *ipaDL99P*. A. Exponential leakage. Samples were collected as described in *Experimental procedures* and Western-blotted with the indicated antibodies. B. Protein secretion in response to Congo red (CR) induction. Samples were collected described in *Experimental procedures*, Silver-stained (top panel) and Western-blotted with the indicated antibodies (bottom panels). C. Total protein expression levels. Samples were collected as described in *Experimental procedures* and Western-blotted with the indicated antibodies. The complemented strain Δ*mxiC/mxiC*^+^ was grown with 25 μM IPTG, while the Δ*mxiC/mxiC(E201K, E276K, E293K)* mutant was grown with 10 μM IPTG because of its higher *mxiC* expression level. Results shown are representative of at least two independent experiments.

Intracytoplasmic MxiC is required for secretion of translocator proteins after activation (Martinez-Argudo and Blocker, 2010). Intriguingly, we generated a mutant in *mxiC* with a very similar phenotype to the *ipaDL99P* mutant: *mxiC(E201K, E276K, E293K)* is a triple mutant in a conserved negatively charged patch on the surface of MxiC that has been suggested to be involved in protein interactions (Deane *et al*., 2008). This mutant also secretes IpaB, IpaC, MxiC and effector proteins in non-inducing conditions. Furthermore, it is also impaired in IpaD secretion after induction, but not affected in induction of any of the other proteins (Fig 6 and not shown). Our results suggest that both the *ipaDL99P* and *mxiC(E201K, E276K, E293K)* mutations cause premature activation of secretion.

**Figure 7:**
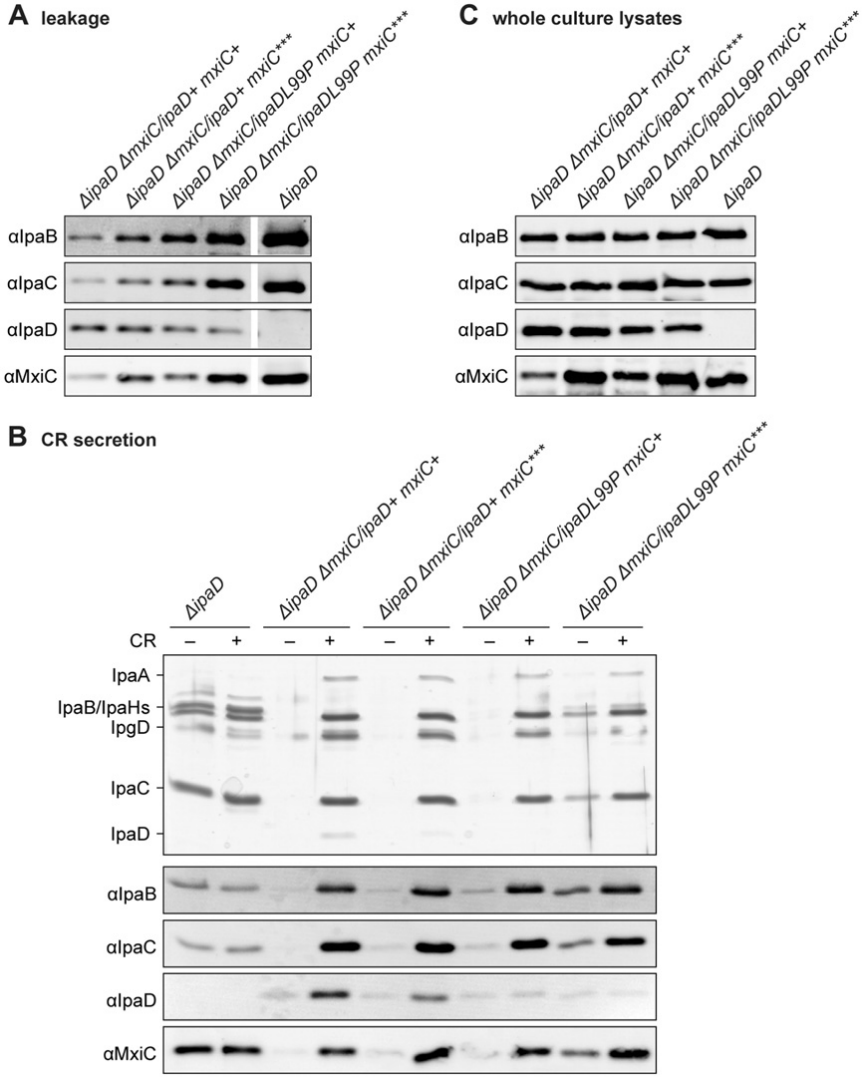
IpaD and MxiC are both involved in the regulation of the mechanism that prevents secretion. A. Exponential leakage of the Δ*ipaD* mutant and a Δ*ipaD* Δ*mxiC* double mutant complemented with *ipaD* and *mxiC* containing plasmids. Samples were collected as described in *Experimental procedures* and Western-blotted with the indicated antibodies. All samples detected with the same antibody were analysed on the same gel. B. Protein secretion in response to Congo red (CR) induction. Samples were collected as described in *Experimental procedures*, Silver-stained (top panel) and Western-blotted with the designated antibodies (bottom panels). C. Total protein expression levels. Samples were collected as described in *Experimental procedures* and Western-blotted with the indicated antibodies. Bacteria were grown with 1% arabinose and 25 μM IPTG as required for expression of *ipaD* and *mxiC* respectively. Mutant *mxiC(E201K, E276K, E293K)* is abbreviated mxiC***. Results shown are representative of at least two independent experiments.

When IpaDL99P and MxiC(E201K, E276K, E293K) were expressed together, we observed an increase in secretion under non-induced conditions in comparison to the single mutants (Fig 7). These results strongly suggest that as MxiC, IpaD is involved in the mechanism that controls secretion.

### Premature secretion in the *ipaDL99P* mutant is MxiC-dependent

**Figure 8:**
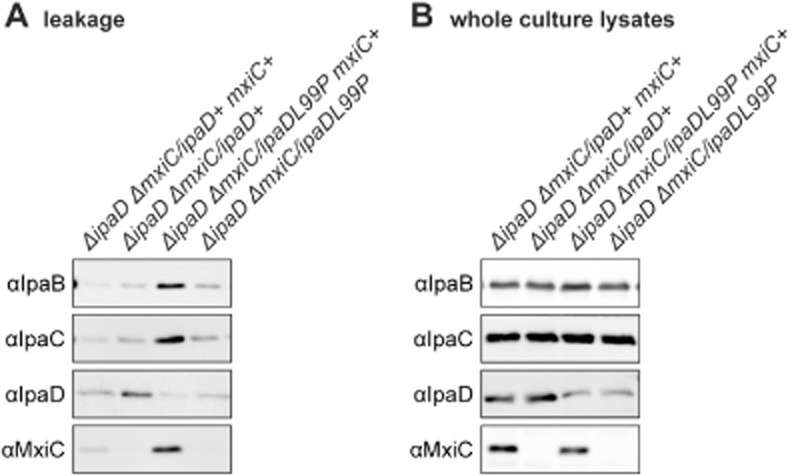
Premature secretion in *ipaDL99P* is MxiC-dependent. A. Exponential leakage of a Δ*ipaD* Δ*mxiC* double mutant complemented with either *ipaD* or *ipaDL99P* alone or additionally with *mxiC*. Samples were collected as described in *Experimental procedures* and Western-blotted with the indicated antibodies. B. Total protein expression levels. Samples were collected as described in *Experimental procedures* and Western-blotted with the indicated antibodies. Bacteria were grown with 1% arabinose and 25 μM IPTG as required for expression of *ipaD* and *mxiC* respectively. Results shown are representative of at least two independent experiments.

We thus wondered whether IpaD is involved in the same activation route as MxiC or whether it has an independent role in controlling secretion. If the former was true, one would expect the *ipaDL99P* mutant to require MxiC for premature secretion. However, if IpaD was directly affecting secretion of the translocators IpaB and IpaC, *ipaDL99P* should lead to MxiC-independent constitutive secretion. To distinguish between these possibilities, we analysed whether IpaB and IpaC were secreted from a Δ*ipaD* Δ*mxiC/ipaDL99P* strain under non-inducing conditions (Fig 8). We found premature secretion in the Δ*ipaD* Δ*mxiC/ipaDL99P* strain was reduced in comparison to *ipaDL99P* alone. Thus, premature secretion of IpaB and IpaC in *ipaDL99P* is dependent on the presence of MxiC.

## Discussion

Type III secretion systems are activated by host cell contact which we proposed leads to a conformational change in the needle tip complex that triggers secretion from the cytoplasm (Veenendaal *et al*., 2007; Blocker *et al*., 2008; Martinez-Argudo and Blocker, 2010). To investigate the role of the major component of the tip complex IpaD in transduction of the activation signal into the cytoplasm, we used a genetic screen to identify *ipaD* mutants unable to secrete after induction by CR. All previous mutations described in IpaD are silent or lead to at least partial loss-of-function and hence constitutive secretion (Picking *et al*., 2005; Barta *et al*., 2012b; Epler *et al*., 2012). Here we describe several mutations in *ipaD* that lead to separate defects in secretion activation (Class I and Class II) and use them to conclude that IpaD has two roles in this process.

### IpaD is involved in signal transduction from the needle tip

All our results suggest that Class II *ipaD* mutants are affected in signalling from the tip complex. Their very identification supports the existence of at least two functional states of the needle tip. All Class II mutants have wild-type-like needle tip complex composition and show a decrease in protein secretion after induction by CR. Furthermore, the defect in secretion of IpaD Class II mutants is not due to their inability to be secreted by the T3SS or to a lack of regulation by MxiC. Finally, combining two ‘strong’ Class IIa mutations generates a non-inducible mutant *ipaD(N186Y, K291I)*.

The ability of the mutants to be induced by CR correlates with their ability to invade HeLa cells: all Class II mutants except for *ipaDN292Y* showed significant decreases in invasion. Therefore, overall our genetic screen yields physiologically meaningful results and supports the notion that CR and host cells are sensed similarly, although the mechanism of sensing remains unknown. It is unclear why the decrease in CR inducibility and the ability to invade host cells do not correlate in mutant *ipaDN292Y*. There are a number of exceptions to the correlation between CR sensing and the ability to invade: three point mutants in *mxiH* are CR inducible but only poorly invasive (Kenjale *et al*., 2005). A reverse correlation was observed in *ipaB*Δ*3*, which is barely inducible by CR, does not perform contact-haemolysis (an assay for the ability to insert the translocon into host membranes) but is still invasive (Roehrich *et al*., 2010). Thus, even though these phenomena are clearly related, their detailed molecular mechanisms are not exactly the same. To understand the cause of these differences, we will need to understand the activation mechanism in molecular detail.

In the constitutively ‘on’ background *ipaB*Δ*3* (Roehrich *et al*., 2010), Class II *ipaD* mutants behave as constitutive secretors indicating that signal transduction is unaffected by these mutations if the tip is already activated by a conformational change in IpaB. This supports the proposal that both IpaD and IpaB are involved in initiation of signal transduction (Veenendaal *et al*., 2007).

### Mutations that affect signalling from the needle tip are located in or near the IpaD C-terminal helix

**Figure 9:**
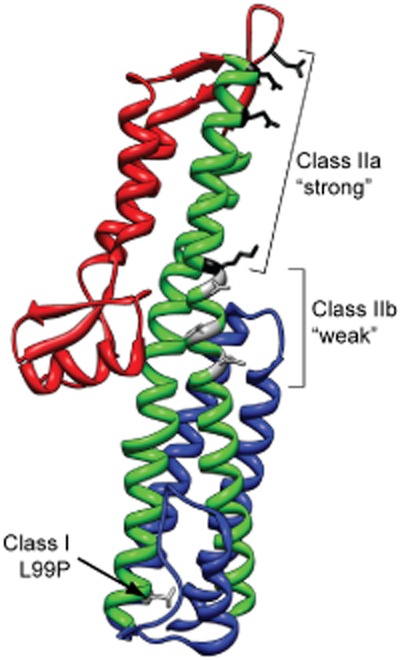
Mapping of identified mutations on the IpaD structure. Mapping of identified mutations on the IpaD structure (PDB entry 2J0O chain A, Johnson *et al*., 28). The N-terminal domain is coloured in blue (residues 40–130), the C-terminal globular domain is coloured in red (residues 177–271) and the coiled coil domain is coloured in green (residues 131–176 and 272–321). Mutated amino acids are shown as stick models, residue L99 (mutated in the Class I mutant) is shown in white, residues mutated in the ‘strong’ Class IIa mutants (N186, N273, Q277 and K291) are shown in black and residues mutated in the ‘weak’ Class IIb (N292, T296, Q299) are shown in grey.

Class II mutations lie in the top of the C-terminal helix (Fig 9) or on that face of the molecule (N186Y). As they all clustered in one part of the molecule, it is unlikely that we have overlooked another major patch of mutants in our screen. In all cases polar or charged amino acids are mutated into hydrophobic or aromatic residues. Intriguingly, the mutations that cause a stronger phenotype (N186Y, N273I + Q277L, K291I) line one side and the ones causing a weaker phenotype (N292Y, T296I, Q299L) line another side of the helix. The C-terminal part of the needle-associated proteins is required for assembly and needle tip binding: mutants containing short C-terminal deletions in the needle protein MxiH fail to produce needles (Kenjale *et al*., 2005; Fujii *et al*., 2012) and similar deletions in IpaB or IpaD reduce or abolish binding to the needle tip (Espina *et al*., 2006; Veenendaal *et al*., 2007; Roehrich *et al*., 2010). Indeed, no mutations were found further down the C-terminal helix of IpaD (residues 300 to 327; 328 to 332 were contained in our PCR primer). Mutations there might also prevent needle binding, leading to constitutive secretion. Such loss-of-function *ipaD* mutants are bright red on CR plates and were excluded in our screen. Deletion of the C-terminal three amino acids of IpaB also leads to a mutant that is constitutively ‘on’ (Roehrich *et al*., 2010). Therefore, the activation signal may be transmitted from the needle tip through the C-terminal helices of IpaD and IpaB. However, structural evidence for the importance of their C-terminal helices in signalling is presently unclear (Chatterjee *et al*., 2011; Lunelli *et al*., 2011; Rathinavelan *et al*., 2011; Barta *et al*., 2012b; Epler *et al*., 2012). Therefore, any conformational change that might or might not be occurring in Class II mutants within the tip complex remains to be established.

### IpaD secretion upon induction may be only regulatory

Class I mutant *ipaDL99P* was specifically affected in induction of IpaD secretion in response to CR induction, but not in secretion of the hydrophobic translocators IpaB and IpaC, MxiC or effectors. It is thus unlikely that this mutant is affected in generation or transduction of the activation signal.

Mutant *mxiC(E201K, E276K, E293K)* shows the same phenotype as *ipaDL99P*. To our knowledge, these are the first mutants that show a different phenotype for secretion of the hydrophilic and hydrophobic translocators. These mutants thus allowed analysis of IpaD's hypothetical extracellular role after induction (Johnson *et al*., 2007). Neither mutant shows a defect in invasion, nor is *mxiC(E201K, E276K, E293K)* affected in survival within (not shown), HeLa cells. Thus, either IpaD secretion is not as reduced *in vivo* as it is *in vitro* or IpaD does not have a specific role in host cell invasion besides being the scaffold for translocon formation at the needle tip. Its secretion upon host cell contact might thus serve a regulatory function only: to remove IpaD from the cytoplasm.

### IpaD has an intracellular role in the same pathway as MxiC

The Class I mutant *ipaDL99P* showed increased secretion of IpaB, IpaC and MxiC under non-inducing conditions, while its expression was reduced in comparison to the complemented strain. The IpaD N-terminal domain was proposed to act as an intramolecular chaperone (Johnson *et al*., 2007; blue domain in Fig 9). Leucine 99 lies in the helix that connects this domain to the central coiled coil of IpaD. Thus, its mutation to proline might affect the conformation of this domain, leading to reduced protein stability. Even though decreased IpaD levels lead to constitutive secretion, premature secretion in *ipaDL99P* is not due to decreased expression, but to a specific effect of the mutation. That we found the same secretion phenotype in a *mxiC(E201K, E276K, E293K)* mutant, which has normal levels of IpaD, further supports this. Interestingly, deletions in the IpaD N-terminal domain (*ipaD*Δ*41–80* and *ipaD*Δ*81–120*; Picking *et al*., 2005), which partially bind the needle tip (Veenendaal *et al*., 2007), secrete MxiC, IpaB and IpaC prematurely (not shown) like *ipaDL99P*. In *Shigella* and *Salmonella* deletion of IpaD or its homologue leads to constitutive secretion (Menard *et al*., 1994; Kaniga *et al*., 1995). In *Yersinia* and *Pseudomonas* the chaperone is a separate polypeptide (LcrG/PcrG; Lee *et al*., 2010; Sato and Frank, 2011) and its deletion has the same effect (DeBord *et al*., 2001; Lee *et al*., 2010). Therefore, a regulatory role of IpaD is probably connected to this domain. As T3SS chaperones are rarely secreted, this further suggests that this regulatory function operates intrabacterially. We cannot confirm the presence of an intrabacterial role of IpaD in type III secretion regulation directly by analysing non-secretable mutants as these are unable to form an external tip complex and are hence deregulated for secretion (Veenendaal *et al*., 2007).

IpaDL99P is also leaked and secreted after CR induction at lower levels than wild-type IpaD. This is not an intrinsic defect of the mutant, but a regulatory defect as the mutant protein is constitutively secreted in a Δ*ipaB* mutant. While we cannot exclude that the decreased IpaD secretion itself is the cause of premature secretion in this mutant, it is more likely to be caused by the regulatory defect as the *mxiC(E201K, E276K, E293K)* mutant has the same general phenotype, but leaks normal levels of IpaD.

Indeed, our results suggest that secretion is prematurely activated in both the *ipaDL99P* and *mxiC(E201K, E276K, E293K)* mutants. In a combined mutant, premature secretion is further increased. We also found that presence of MxiC is necessary for premature secretion in the *ipaDL99P* mutant. This indicates that in these mutants secretion is activated from within the bacterial cytoplasm. Altogether, our results indicate that MxiC and IpaD act in the same pathway to regulate secretion in response to the absence or presence of the activation signal. Therefore, our findings also suggest a link between LcrG/PcrG and the MxiC homologues YopN/PopN in secretion activation.

We previously suggested that in *Shigella*, translocator secretion is prevented by a ‘repressor mechanism’ (R), that is put in place when the tip is finished and which is absent in Δ*ipaD* and Δ*ipaB* mutants. We have also shown that cytoplasmic MxiC is required for induction of translocator secretion (Martinez-Argudo and Blocker, 2010). We thus now propose that the *mxiC(E201K, E276K, E293K)* mutant has lost the ability to promote IpaD secretion but gained the ability to activate secretion of hydrophobic translocators and itself. In addition, the isolation and analysis of the *ipaDL99P* mutant now suggests that IpaD is directly involved in regulating secretion from the bacterial cytoplasm. It is plausible that the cytoplasmic function of IpaD is to prevent premature secretion before reception of the activation signal, i.e. that IpaD is a repressor of translocator secretion. In this case *ipaDL99P* would be a loss-of-function mutant as it is unable to fulfil its repression function. Such secreted repressors coupling induction to hierarchical secretion and expression are common in T3SSs (Brutinel and Yahr, 2008; Chevance and Hughes, 2008). Alternatively, IpaD could act as a co-activator of translocator secretion alongside MxiC. In that case, IpaDL99P would fulfil this function even though it should only do so after activation, thus it would be a gain-of-function mutant for translocator secretion. Either way, it is now clear that MxiC and IpaD are both involved in regulation of an early step in secretion activation within the bacterial cytoplasm.

### Conclusion

We demonstrate here that IpaD controls secretion both by regulating the functional state of a transmembrane macromolecular machine and as part of a signal transduction pathway that is dependent on cytoplasmic proteins. By genetically separating these activities (in Class I vs. Class II mutants), we demonstrate that one protein pool acts indirectly on another pool of the same protein to orchestrate T3SS activation. To our knowledge, this has rarely been reported so far, possibly because such functions are difficult to separate. It may thus represent a novel regulatory paradigm.

## Experimental procedures

### Bacterial strains, plasmids and primers

Table 2 lists the strains and plasmids used in this study. *S. flexneri* strains were grown in Trypticase Soy Broth (Becton Dickinson) at 37°C with the appropriate antibiotics at the following final concentrations: ampicillin 100 μg ml^−1^, kanamycin 50 μg ml^−1^, tetracycline 5 μg ml^−1^, chloramphenicol 10 μg ml^−1^. For the inducers IPTG and arabinose we used the final concentrations indicated in the figure legends. Table S1 lists the primers used in this study.

**Table 2:** *Shigella flexneri* strains and plasmids used in this study

Strain name	Description	Reference
Wild-type	Wild-type M90T, serotype 5a	Sansonetti *et al*. (1982)
SF620 or Δ*ipaB*	Δ*ipaB::aphA-3* mutant	Menard *et al*. (1993)
SF622 or Δ*ipaD*	Δ*ipaD::aphA-3* mutant	Menard *et al*. (1993)
Δ*mxiC*	Δ*mxiC::tetRA* mutant	Martinez-Argudo and Blocker (2010)
Δ*ipaB* Δ*ipaD*	Double mutant Δ*ipaB::aphA-3* Δ*ipaD::tetRA*	This study
Δ*ipaD* Δ*mxiC*	Double mutant Δ*ipaD::aphA-3* Δ*mxiC::tetRA*	This study
Δ*ipaD/ipaD*^+^	Δ*ipaD* mutant/pUC18_ipaD	Picking *et al*. (2005)
*ipaD(L99P, N255D)*	Mutant isolated from an *ipaD* mutant library	This study
*ipaDL99P*	Δ*ipaD* mutant/pIMA230	This study
*ipaDN186Y*	Δ*ipaD* mutant/pIMA231 (isolated from an *ipaD* mutant library)	This study
*ipaD(N273I, Q277L)*	Δ*ipaD* mutant/pIMA232 (isolated from an *ipaD* mutant library)	This study
*ipaDK291I*	Δ*ipaD* mutant/pIMA233 (isolated from an *ipaD* mutant library)	This study
*ipaDN292Y*	Δ*ipaD* mutant/pIMA234 (isolated from an *ipaD* mutant library)	This study
*ipaDT296I*	Δ*ipaD* mutant/pIMA235 (isolated from an *ipaD* mutant library)	This study
*ipaD(Y104L, Q299L)*	Mutant isolated from an *ipaD* mutant library	This study
*ipaD/ipaDQ299L*	Δ*ipaD* mutant/pIMA236	This study
*ipaD/ipaD(L99P, K291I)*	Δ*ipaD* mutant/pIMA238	This study
*ipaD/ipaD(N186Y, K291I)*	Δ*ipaD* mutant/pIMA237	This study
Δ*ipaB* Δ*ipaD/ipaD*^+^	Δ*ipaB* Δ*ipaD* double mutant/pUC18_ipaD	This study
Δ*ipaB ipaDL99P*	Δ*ipaB* Δ*ipaD* double mutant/pIMA230	This study
*ipaB*Δ*3* Δ*ipaD/ipaD*^+^	Δ*ipaB* Δ*ipaD* double mutant/pIMA241	This study
*ipaB*Δ*3 ipaDL99P*	Δ*ipaB* Δ*ipaD* double mutant/pIMA242	This study
Δ*mxiC/mxiC*^+^	Δ*mxiC* mutant/pIMA227	This study
*mxiC(E201K, E276K, E293K)*	Δ*mxiC* mutant/pDR67	This study
Δ*ipaD* Δ*mxiC/ipaD*^+^	Δ*ipaD* Δ*mxiC* double mutant/pIMA243	This study
Δ*ipaD* Δ*mxiC/ipaDL99P*	Δ*ipaD* Δ*mxiC* double mutant/pIMA244	This study
Δ*ipaD* Δ*mxiC/ipaD*^+^ *mxiC*^+^	Δ*ipaD* Δ*mxiC* double mutant/pIMA243 and pIMA227	This study
Δ*ipaD* Δ*mxiC/ipaDL99P mxiC*^+^	Δ*ipaD* Δ*mxiC* double mutant/pIMA244 and pIMA227	This study
Δ*ipaD* Δ*mxiC/ipaD*^+^ *mxiC(E201K, E276K, E293K)*	Δ*ipaD* Δ*mxiC* double mutant/pIMA243 and pDR67	This study
Δ*ipaD* Δ*mxiC/ipaDL99P mxiC(E201K, E276K, E293K)*	Δ*ipaD* Δ*mxiC* double mutant/pIMA244 and pDR67	This study

Plasmid	Description	Reference
pUC18_ipaD	*ipaD* gene cloned into a modified pUC18	Picking *et al*. (2005)
pDR2	pUC19*_ipaB*Δ*3*, *ipaB* C-terminal 3aas truncation	Roehrich *et al*. (2010)
pDR67	pACT3_*mxiC(E201K, E276K, E293K)*	This study
pIMA212	pACT3_*mxiH*	This study
pIMA227	pACT3_*mxiC*	Martinez-Argudo and Blocker (2010)
pIMA230	pUC18_*ipaDL99P*	This study
pIMA231	pUC18_*ipaDN186Y*	This study
pIMA232	pUC18_*ipaD(N273I, Q277L)*	This study
pIMA233	pUC18_*ipaDK291I*	This study
pIMA234	pUC18_*ipaDN292Y*	This study
pIMA235	pUC18_*ipaDT296I*	This study
pIMA236	pUC18_*ipaDQ299L*	This study
pIMA237	pUC18_*ipaD(N186Y, K291I)*	This study
pIMA238	pUC18_*ipaD(L99P, K291I)*	This study
pIMA239	pUC18_*ipaD(N292Y, T296I, Q299L)*	This study
pIMA241	pUC19_*ipaB*Δ*3 ipaD*	This study
pIMA242	pUC19_*ipaB*Δ*3 ipaDL99P*	This study
pIMA243	pBADMyc_HisA_*ipaD*	This study
pIMA244	pBADMyc_HisA_*ipaDL99P*	This study

### Construction of the *ipaD* mutant libraries

PCR mutagenesis was carried out with *Taq* DNA polymerase (New England Biolabs) by using standard or error prone (3 mM Mg^2+^ and 4 mM Mg^2+^) reaction conditions using primers ipaD_NdeI and ipaD_BamHI and pUC18_*ipaD* as template. PCR fragments were purified, digested with NdeI and BamHI and cloned into plasmid pUC18_*ipaD* (Picking *et al*., 2005) digested with the same enzymes. Ligation mixtures were electroporated into DH5α. The transformation mixture was incubated for 16 h, and plasmid DNA was then extracted and kept at −20°C.

### Screening of the *ipaD* mutant libraries

To identify non-inducible mutants, the DNA from each of the three mutant libraries was electroporated into *S. flexneri* Δ*ipaD* mutant and screened for white colonies on TCSB agar plates containing 100 μg ml^−1^ CR (Serva). Putative ‘white’ colonies were selected and their plasmids isolated and retransformed into the Δ*ipaD* mutant to ensure the white colour was not due to loss-of-function mutations elsewhere within the T3SS-encoding operons. Candidate plasmids were sequenced to identify the mutation(s) responsible for the mutant phenotype.

### Construction of non-polar knockout mutant strains

The Δ*ipaB* Δ*ipaD* mutant was generated by using the Lambda Red system as described by Datsenko and Wanner (2000). Briefly, to replace the wild-type *ipaD* gene, a tetracycline resistance cassette with 50 bp flanking regions homologous to the *ipaD* gene was amplified from strain TH2788 (Frye *et al*., 2006) using the primers ipaD_KO_tetF and ipaD_KO_tetR (Table S1). The *ipaD* gene was exchanged for a tetracycline cassette in the single mutant strain SF620 (Δ*ipaB*) following the procedure previously described (Martinez-Argudo and Blocker, 2010). To generate strain Δ*ipaD* Δ*mxiC*, the same procedure was used to exchange the *mxiC* gene for a tetracycline cassette in the mutant strain SF622 (Δ*ipaD*) using the primers mxiC_KO_tetF and mxiC_KO_tetR (Table S1). All gene replacements were confirmed by sequencing.

### Construction of plasmids

To express the *ipaD* mutations in combination with *ipaB*Δ*3*, a plasmid encoding both *ipaB*Δ*3* and *ipaD* was constructed. The *ipaD* or *ipaDL99P* genes were amplified by PCR using primers ipaD_PstI_F and ipaD_BamHI (Table S1) and pUC18_*ipaD* or pIMA230 (pUC18_*ipaDL99P*) as template. PCR products were purified, digested with PstI and BamHI and cloned into pDR2 (pUC19_*ipaB*Δ*3*) digested with the same enzymes, giving rise to plasmids pIMA241 and pIMA242 respectively.

To construct the double *ipaD* mutants, *ipaD(L99P, K291Y)* and *ipaD(N186Y, K291Y)*, a HindIII-BamHI fragment from plasmid pIMA233 (*ipaDK291I*) was cloned into pIMA230 and pIMA231, digested with the same enzymes, giving rise to pIMA238 and pIMA237 respectively.

Plasmid pIMA239 (*ipaD(N292Y, T296I, Q299L)*) was made by two-step PCR. The first step consisted of two PCR reactions, one carried out with primer ipaD_NdeI and reverse primer ipaD_tripleR containing the three desired mutations and the other with forward primer ipaD_tripleF containing the mutations and reverse primer ipaD_BamHI (Table S1). The two PCR fragments were used as a template for the second PCR using primers ipaD_NdeI and ipaD_BamHI. The PCR product was purified, digested with HindIII and BamHI and cloned into pUC19_*ipaD* digested with the same enzymes.

To express *ipaD* and *ipaDL99P* from an inducible plasmid we chose plasmid pBAD/*Myc*_HisA (Invitrogen). The *ipaD* or *ipaDL99P* genes were amplified by PCR using primers ipaD_NcoI_F and ipaD_PstI_rev (Table S1) and pUC18_*ipaD* or pIMA230 (pUC18_*ipaDL99P*) as template. PCR products were purified, digested with NcoI and PstI and cloned into pBAD/*Myc*_HisA digested with the same enzymes, giving rise to plasmids pIMA243 and pIMA244 respectively.

To overexpress the needle protein MxiH, the *mxiH* gene was amplified by PCR using the *Shigella* virulence plasmid pWR100 (Buchrieser *et al*., 2000) as template and primers *mxiH*_RBS and *mxiH*_HindIII (Table S1). The PCR product was purified, digested with SacI and HindIII, and cloned into the IPTG inducible plasmid pACT3 (Dykxhoorn *et al*., 1996) giving rise to pIMA212.

Plasmid pDR67 (*mxiC(E201K, E276K, E293K)*) was made by three subsequent PCR steps. In the first step four PCR fragments were generated using pWR100 as a template and primers mxiC_SacI and mxiC_E201K_rev, mxiC_E201K_for and mxiC_E276K_rev, mxiC_E276K_for and mxiC_E293K_rev and mxiC_E293K_for and mxiC_BamHI (Table S1). In the second step the first two and the last two fragments were combined using primers mxiC_SacI and mxiC_E276K_rev or mxiC_E276K_for and mxiC_BamHI respectively. The final PCR product was generated by combining these fragments using primers mxiC_SacI and mxiC_BamHI. It was purified and digested with SacI and BamHI. The digested fragment was cloned into pACT3 (Dykxhoorn *et al*., 1996) restricted with the same enzymes.

All plasmids were verified by sequencing.

### Analysis of protein synthesis and secretion

#### Total level of protein expression

*Shigella flexneri* strains were grown at 37°C until mid-exponential growth (OD_600_ ≍ 1) was reached. Samples of the cultures, representing the total protein fraction, were denatured in Laemmli sample buffer. Samples from equivalent cell numbers were separated by SDS-PAGE and used for Western blot analysis.

#### Exponential leakage

*Shigella flexneri* strains were grown until OD_600_ ≍ 1 (mid-exponential phase). Cultures were centrifuged at 15 000 *g* for 10 min at 4°C and supernatants from equivalent cell numbers were denatured in Laemmli sample buffer, subjected to SDS gel electrophoresis and Silver-stained (Silver Xpress kit, Invitrogen) or used for Western blot analysis.

#### Congo red induction

Bacteria collected during mid-exponential growth (OD_600_ ≍ 1) were resuspended at OD_600_ = 5 in phosphate-buffered saline (PBS). CR (Serva) was added at a final concentration of 200 μg ml^−1^ to induce T3SS activity. After incubation at 37°C for 15 min, the samples were centrifuged at 15 000 *g* for 10 min at 4°C and the supernatants were denatured, separated by SDS-PAGE and Silver-stained (Silver Xpress kit, Invitrogen) or used for Western blot analysis.

### Western blot analysis

For Western blot analysis, proteins separated by SDS-PAGE were transferred onto PVDF membrane (Immobilon FL, Millipore) and hybridized with the following primary antibodies: IpaB, IpaC, IpaD and MxiC (see Martinez-Argudo and Blocker, 2010 for details). Fluorescent secondary antibodies (goat anti-rabbit-Alexa680, Invitrogen; goat anti-rabbit-DyLight800 and goat anti-mouse-DyLight800, Pierce) were visualized and quantified using an Odyssey infrared imaging system (Li-Cor). For quantifications, standard curves obtained by serial dilution of a sample from the complemented strain were run on the same gel to control for linearity of the signal. Numbers are averages of two independent experiments.

### Needle preparations

To analyse tip complex compositions we isolate needles sheared from the surface of bacteria that overexpress the needle protein MxiH and hence make very long needles (as normal length needles are too short to be efficiently sheared off). Needles were purified from non-activated strains containing plasmid pIMA212 (pACT3_*mxiH*) as described in Veenendaal *et al*. (2007). Briefly, 250 ml of cultures were grown in TCSB overnight with 200 μM IPTG to induce overproduction of MxiH from plasmid pIMA212. Needles were sheared off, separated by differential centrifugation and precipitation and further purified by size exclusion chromatography and fractions containing needles were pooled. Samples were denatured in Laemmli sample buffer, separated by SDS-PAGE and Silver-stained (Silver Xpress kit, Invitrogen) to normalize the amount of MxiH prior to Western blotting to detect IpaB and IpaD.

### Invasion assays

*Shigella flexneri* invasion of HeLa cells was assessed with a gentamicin protection assay as described previously (Roehrich *et al*., 2010). Briefly, HeLa cells were seeded into 24-well plates and infected with bacteria grown to mid-exponential phase at a multiplicity of infection of 100. After centrifugation for 10 min at 900 *g*, the infected cells were incubated for 30 min at 37°C. Cells were then washed with PBS and gentamicin was added to kill extracellular bacteria. After further incubation for 1 h, cells were lysed in 0.1% Triton X-100; a dilution series was prepared and plated on LB agar plates. Colonies were counted the next day.
