# Supplementary material for: Shigella IpaD has a dual role: signal transduction from the type III secretion system needle tip and intracellular secretion regulation
Source: Mol Microbiol. 2013 Jan 11;87(3):690–706. doi: 10.1111/mmi.12124 (PMC3575693; doi:10.1111/mmi.12124)
Supplement: Supplementary file 1 [file mmi0087-0690-sd1.pdf]

Supporting information for

Shigella IpaD has a Dual Role: Signal Transduction from the T3SS Needle Tip and Intracellular Secretion Regulation

A. Dorothea Roehrich[1], Enora Guillossou[1], Ariel J. Blocker[1,2]* and Isabel Martinez-Argudo[1#]

[1]School of Cellular & Molecular Medicine, University of Bristol, BS8 1TD, United Kingdom

[2]School of Biochemistry, University of Bristol, BS8 1TD, United Kingdom

[#]Present address: Área de Genética, Facultad de Ciencias Ambientales y Bioquímica, Campus Tecnológico de la Fábrica de Armas, Universidad de Castilla-La Mancha, Avda. Carlos III, s/n, E-45071, Toledo, Spain

*Corresponding author: Schools of Cellular & Molecular Medicine and Biochemistry, University of Bristol, University Walk, BS8 1TD, United Kingdom

Tel: +44-1173-312-063; Fax: +44-1173-312-091; E-mail: ariel.blocker@bristol.ac.uk

Supplementary figures

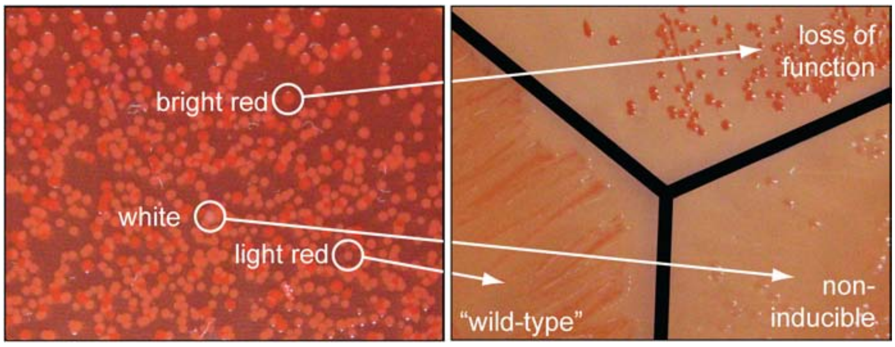

Figure S1. Non-inducible *ipaD* mutants are white on Congo red agar plates.

An *ipaD* library was plated on agar plates containing 100 µg/ml CR and white colonies were picked and restreaked on fresh plates to confirm their phenotype. Wild-type *Shigella* is light red on these plates while constitutive secreters like the Δ*ipaD* strain or loss-of-function mutants are bright red, probably because CR stains effector proteins. Non-inducible mutants are white.

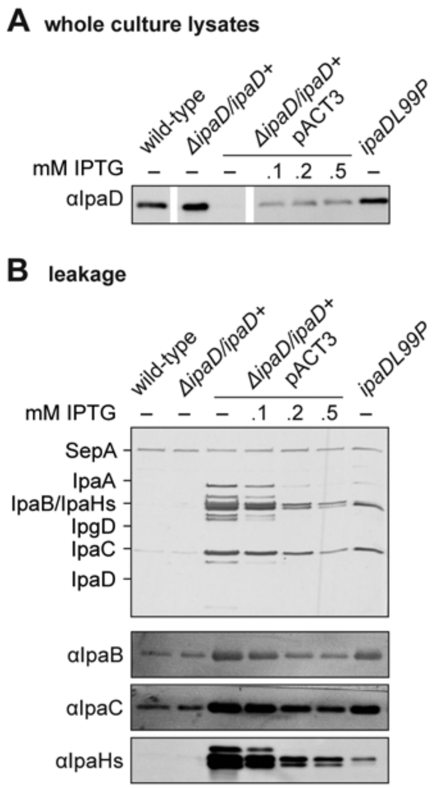

Figure S2. Premature secretion in *ipaDL99P* is not due to lower expression levels.

To analyse the phenotype of a strain expressing less than wild-type levels of IpaD, expression of *ipaD* in the complemented strain Δ*ipaD/ipaD*$^+$ (which contains *ipaD* on a pUC plasmid) was repressed in trans by addition of the *lac* repressor from pACT3 and then derepressed by IPTG induction using no (lane –), 0.1 mM, 0.2 mM or 0.5 mM IPTG.

(A) Total protein expression levels. Samples were collected as described in Experimental procedures and Western-blotted with an antibody against IpaD. IpaD expression in a Δ*ipaD/ipaD*$^+$ strain containing pACT3 could not be significantly increased beyond the levels detected after induction with 0.2 mM IPTG and never reached wild-type levels.

(B) Exponential leakage. Samples were collected as described in Experimental procedures, Silver-stained (top panel) and Western-blotted with the indicated antibodies (bottom panels). Very low levels of IpaD as observed in Δ*ipaD/ipaD*$^+$ pACT3 induced with 0.5 mM IPTG are sufficient to largely prevent premature secretion in comparison to a strain lacking IpaD expression (uninduced Δ*ipaD/ipaD*$^+$ pACT3, lane –). Furthermore, the protein pattern

observed in Δ*ipaD/ipaD*$^+$ pACT3 induced with 0.5 mM IPTG differs from the pattern in *ipaDL99P*: the latter secretes much higher levels of translocators IpaB and IpaC, and lower levels of IpaH (late effector), indicating that this phenotype is a specific effect of the mutation and not merely an expression defect.

Results shown are representative of at least two independent experiments.

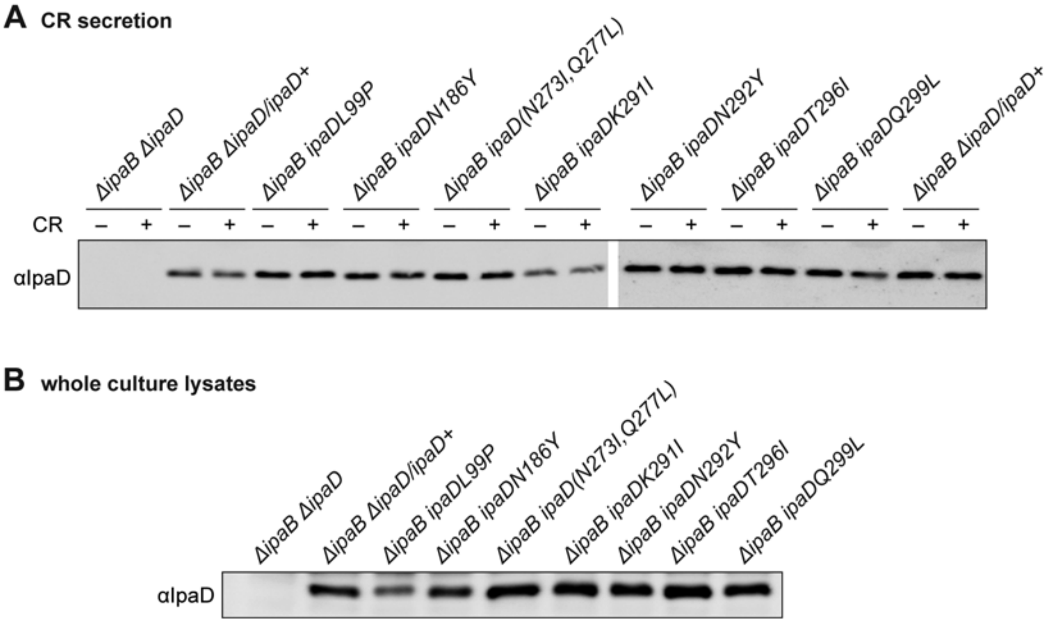

Figure S3. The IpaD mutant proteins are secreted in a constitutive secretor background.

(A) Secretion of IpaD after CR induction by a Δ*ipaB* Δ*ipaD* double mutant complemented with the indicated *ipaD* plasmids. Samples were collected as described in Experimental procedures and Western-blotted with an antibody against IpaD.

(B) Total protein expression levels. Samples were collected as described in Experimental procedures and Western-blotted with an antibody against IpaD.

Results shown are representative of at least two independent experiments.

Supplementary tables

Table S1. Primers used in this study.

Primer	Sequence*
ipaD_BamHI	CGCGGATCCTCAGAAATGGAGAAAAAG
ipaD_KO_tetF	AATAATATATGGCTCTTCCTGTAAGGAAATAACCATGAATATAACAACTTAAGACCCACTTTCA
ipaD_KO_tetR	TATTATTTACATTATGCATGGCGCACCTCAGAAATGGAGAAACATATGAATATCCTCCTTACTAAGCACTTGTCTCCTG
ipaD_NcoI	CCATGCCATGGATATAACAACTCTGACTAATAG
ipaD_NdeI	GATTACGAATTCCATATGAATATAACAACTC
ipaD_PstI_F	AAAACTGCAGTAAGGAAATAACCATGAATATAACAACTCTGACTAATAG
ipaD_PstI_rev	AAAACTGCAGTCAGAAATGGAGAAAAAGTTTATC
ipaD_tripleF	GAAGATGAAACAATGAAA**TAT**AATCTTCAA**ATT**TTAGTT**CTA**AAATACAGTAATGCCAATAG
ipaD_tripleR	CTATTGGCATTACTGTATTT**TAG**AACTAA**AAT**TTGAAGATT**ATA**TTTCATTGTTTCATCTTC
mxiH_RBS	CGGAATTCGAGCTCCAGGAGGAATTACATATGAGTGTTACAGTACCGAATGATGATTG
mxiH_HindIII	CCATCGATAAGCTTTTATCTGAAGTTTTGAATAATTG
mxiC_BamHI	CGCGGATCCCTGGATCACTTTTATCTCCTGTTATC
mxiC_KO_tetF	CATTGGTTTCATACTTAAATTACTAACTATAAAGTAGGTGATGTATGCTTGATGTTAATTAAGACCCACTTTCA
mxiC_KO_tetR	TGCTTAAGAAAAGACTGGATCACTTTTATCTCCTGTTATCTAGAAAGCTCCATATGAATATCCTCCTTACTAAGCACTTGTCTCCTG
mxiC_SacI	GCACGCGAGCTCAACTATAAAGTAGGTGATGTATGCTTG
mxiC_pwr_KpnI_F	CTAGGGTACCAACTATAAAGTAGGTGATGTATGCTTG
mxiC_SalI_rev	CTAGGTCGACTTATCTAGAAAGCTCTTTCTTGTATG
mxiC_E201K_for	CAACAACAGATCAGTATATA**A**AATGGCTTGGTAATTTTGG
mxiC_E201K_rev	CCAAAATTACCAAGCCATT**T**TATATACTGATCTGTTGTTG
mxiC_E276K_for	CTAAAAGATGGCAGTTTAAGTGCA**A**AGCAGCTATTGCTAAC
mxiC_E276K_rev	GTTAGCAATAGCTGCT**T**TGCACTTAAACTGCCATCTTTTAG
mxiC_E293K_for	TATCCAAGTGAAAGT**A**AGCAAATTCTTACTTCTGTTATAG
mxiC_E293K_rev	TAACAGAAGTAAGAATTTGCT**T**ACTTTCACTTGGATATTG

* Restriction sites are underlined, mismatches in mutagenesis primers are indicated in **bold** letters
